# Persistent and sporadic *Listeria monocytogenes* strains do not differ when growing at 37 °C, in planktonic state, under different food associated stresses or energy sources

**DOI:** 10.1186/s12866-019-1631-3

**Published:** 2019-11-19

**Authors:** Alexander J. Taylor, Matthew J. Stasiewicz

**Affiliations:** Department of Food Science and Human Nutrition, College of Agricultural, Consumer, and Environmental Sciences, University of Illinois at Urbana-Champaign, Urbana, IL 61801 USA

**Keywords:** Persistence, *Listeria monocytogenes*, Defined media, Growth rate, Extrinsic stress

## Abstract

**Background:**

The foodborne pathogen *Listeria monocytogenes* causes the potentially lethal disease listeriosis. Within food-associated environments, *L. monocytogenes* can persist for long periods and increase the risk of contamination by continued presence in processing facilities or other food-associated environments. Most research on phenotyping of persistent *L. monocytogenes*’ has explored biofilm formation and sanitizer resistance, with less data examining persistent *L. monocytogenes*’ phenotypic responses to extrinsic factors, such as variations in osmotic pressure, pH, and energy source availability. It was hypothesized that isolates of persistent strains are able to grow, and grow faster, under a broader range of intrinsic and extrinsic factors compared to closely related isolates of sporadic strains.

**Results:**

To test this hypothesis, 95 isolates (representing 74 isolates of 20 persistent strains and 21 isolates of sporadic strains) from a series of previous studies in retail delis, were grown at 37 °C, in (i) stress conditions: salt (0, 5, and 10% NaCl), pH (5.2, 7.2, and 9.2), and sanitizer (benzalkonium chloride, 0, 2, and 5 μg/mL) and (ii) energy sources: 25 mM glucose, cellobiose, glycogen, fructose, lactose, and sucrose; the original goal was to follow up with low temperature experiments for treatments where significant differences were observed. Growth rate and the ability to grow of 95 isolates were determined using high-throughput, OD_600_, growth curves. All stress conditions reduced growth rates in isolates compared to control (*p* < 0.05). In addition, growth varied by the tested energy sources. In chemically defined, minimal media there was a trend toward more isolates showing growth in all replicates using cellobiose (*p* = 0.052) compared to the control (glucose) and fewer isolates able to grow in glycogen (*p* = 0.02), lactose (*p* = 2.2 × 10^− 16^), and sucrose (*p* = 2.2 × 10^− 16^). Still, at least one isolate was able to consistently grow in every replicate for each energy source.

**Conclusions:**

The central hypothesis was rejected, as there was not a significant difference in growth rate or ability to grow for retail deli isolates of persistent strains compared to sporadic strains for any treatments at 37 °C. Therefore, these data suggest that persistence is likely not determined by a phenotype unique to persistent strains grown at 37 °C and exposed to extrinsic stresses or variation in energy sources.

## Background

### *Listeria monocytogenes*

*Listeria monocytogenes* is a foodborne pathogen that causes listeriosis and is estimated to cause 1600 illnesses and 260 deaths per year in the US [1]. *L. monocytogenes* is found commonly in natural and agricultural soil, water, and animals, where it can contaminate raw food materials directly and be transferred into processing facilities [2]. *L. monocytogenes* can be found on ready-to-eat (RTE) foods, such as produce, soft cheeses, and, relevant to this study, deli meats [3]. In 2003, a risk assessment linked 90% of listeriosis cases in the U.S. to contaminated RTE deli meats [3]. While more recent work also suggests produce is emerging as another high-risk commodity [4–7], listeriosis remains a concern in deli meats [4, 6, 8–11]. Typically, one may find growth niches, or harborage sites, of *Listeria* usually in areas that are difficult to clean, such as drains, condenser coils, cold rooms, or microcracks within bulky, heavy processing equipment [12, 13], as well as some food contact surfaces, such as deli slicers, counters, or cutting boards [14]. When water and organic materials are available in niches, *L. monocytogenes* can not only survive but multiply [15, 16]. Once in the niche, the pathogen may form a biofilm, or become associated with a pre-existing biofilm [17–20]. Biofilms can make it even harder to eliminate the strain as the biofilm physically protects the strain from exposure to bactericidal concentrations of sanitizer [21, 22].

*L. monocytogenes* can also survive and grow pHs as low as 4.7 and as high as 9.2 [23], high salt concentrations (10% w/v) [24], and in the presence of sub-lethal concentrations of antimicrobial solutions or sanitizers (amount varies per sanitizer) [25, 26]. Resistance to these extrinsic stresses likely contribute to its survival in processing environments where pH, osmotic, and sanitizer stresses are common [27–30], and survival represents a risk for cross-contaminating food products produced in those environments.

### Persistence

*L. monocytogenes* can persist in food processing environments for months to decades [23, 31]. Persistent strains represent a continual risk of contamination within a manufacturing or processing environment. For this paper, persistence is defined as the continued presence of a clonal population of bacteria, over time, at a specific location [32], due to long-term survival with or without population growth. That clonal population is a ‘strain’ and when testing for the presence of the bacteria at that location once may collect a specific ‘isolate’ of a persistent strain. Many researchers believe that niches within the food environment [21], biofilm formation [33] including mixed biofilms [34–37], and *L. monocytogenes*’ resistance to sanitizers [38, 39] and other extrinsic stresses, may contribute to strain persistence. While the contribution of niches and biofilm formation have been extensively discussed as components of *L. monocytogenes* persistence, this study will focus on a relative gap in the literature on persistent versus sporadic *L. monocytogenes* phenotypic responses to extrinsic stresses and energy source availability – specifically growth at 37 °C as a rapid screen and proxy for potential persistence ability.

### Relevant stress response phenotypes of persistent strains

There are relatively few reports comparing persistent and sporadic strains for differences in salt and acid tolerance. One recent paper stated that persistent strains from a cheese-processing facility were better adapted than sporadic strains to grow in 2.5, 4, and 8% NaCl and acidic, pH 5, conditions [28]. Another earlier paper compared acid tolerance in 17 persistent to 23 non-persistent strains from three meat-processing plants [12]. No difference was observed in log reduction after the acid stress, but the authors noted that two non-persistent strains were the most acid-sensitive strains. In contrast, there are multiple reports comparing persistent and sporadic strains for differences in response to with benzalkonium chloride (BAC). One research article [40] reported 14 persistent isolates, from two separate pork-processing plants, with BAC resistant genes. These isolates related back to the multilocus sequence typing (MLST) sequence type 121 (ST121) [40], which has been known to be both persistent and contains the BAC resistant transposon Tn*6188* [2, 40]. However, not all isolates of persistent strains contain this transposon or BAC resistant genes [23]. The *bcr*ABC cassette has been attributed to BAC resistance, but not every strain, persistent or non-persistent, contains this, likely, plasmid localized operon [41, 42]. Overall, there is a lack of consistency in the literature on whether persistent strains are more resistant to particular stress conditions compared to sporadic strains from similar sources.

### Classification of persistent strains and relationship to phenotype work

One potential explanation for inconsistency in phenotyping studies’ results is that each study has its own rules to identify persistent and sporadic comparison groups. Persistent strains are typically empirically identified by isolating, on different sampling dates, *L. monocytogenes* that are found to be indistinguishable, or otherwise of the same stain, by the best subtyping method available to the researchers [23]. For example, the source studies for isolates used in this study required indistinguishable isolates to be isolated on at least 3 sampling periods each at least 1 month apart. In particular, many publications [8, 43–45] used pulsed-field gel electrophoresis (PFGE) for subtyping. While PFGE has been the gold standard for assessing genetic relationships between *L. monocytogenes* isolates, this technique has recently been replaced by whole genome sequencing (WGS), which has helped to improve listeriosis outbreak investigations, genotypic subtyping, and allow for other inquiries [46]. As the costs continue to lower, WGS is becoming a viable alternative for distinguishing strains and investigating contamination in food processing plants [47]. WGS has also been used to improve the differentiation of persistent and sporadic strains from retail delis in multiple regions of the US [32]. Yet, the authors’ are unaware of published work comparing phenotypes of persistent and sporadic strains using strains classified by WGS-based methods.

### Motivation and hypothesis

The aim of this study was to compare extrinsic factor phenotypic responses between persistent and sporadic strains of *L. monocytogenes* classified by the best available subtyping methods. To do so, 95 isolates of persistent and sporadic strains collected from a previous, longitudinal study of 30 retail delis across the US. First the isolates were subtyped by PFGE [8]. Then, in a follow-up study, all of the isolates were sequenced and WGS-based methods were used to refine the identification of persistent strains, specifically by a, core-genome, Single Nucleotide Polymorphisms (SNP)-difference metric [32]. From that work, the isolates were reliably classified as persistent or sporadic strains representing 25 putative persistence events (isolates from more than one sampling time forming a well-supported clade) and closely related sporadic strains (from the same genetic clade). For this study, a panel of 95 isolates was assembled representing 74 isolates of 20 persistent strains and 21 isolates of closely related sporadic strains. The panel represented a sample set with the statistical power to rigorously test if isolates of persistent and sporadic strains differ in growth responses (ability to growth, growth rate if able to grow) to osmotic pressure, acidic and alkali conditions, sanitizers, and energy sources. The hypothesis was that if persistent isolates have adapted advantages over closely related, sporadic strains, they would show significantly greater growth rates, or ability to grow, in the presence of these extrinsic stress conditions and energy sources in a high-throughput screening experiment at 37 °C.

## Results and discussion

Ninety-five *L. monocytogenes* isolates, comprised of 74 isolates of 20 persistent strains and 21 sporadic strains, were tested for their growth rates and ability to grow in the presence of extrinsic stress conditions and utilization of energy sources (Table 1). These strains were collected from a previous, longitudinal study in retail delis [8], where persistent strains were identified based on WGS core genome SNP analysis [32].

**Table 1:** Description of treatments for the extrinsic stress and energy source test, including pre-growth, treatment media formulation, and replicates tested

Treatments	Replicates^a^	Pre-growth media	Energy Source	Salt (%)	pH levels	BAC^b^ (μg/mL)
Stress Conditions (tested in nutritive media [BHI])						
Control	3	BHI	Dextrose	0.5	7.20	0
5% NaCl	4	BHI	Dextrose	5	7.20	0
10% NaCl	4	BHI	Dextrose	10	7.20	0
pH of 5.2	3	BHI	Dextrose	0.5	5.20	0
pH of 9.2	3	BHI	Dextrose	0.5	9.20	0
BAC 2 μg/mL	6	BHI	Dextrose	0.5	7.20	2
BAC 5 μg/mL	6	BHI	Dextrose	0.5	7.20	5
Energy Source Utilization (tested in chemically defined media [DM])						
DM Control	3	DM Glucose	Glucose	0.5	6.75	0
DM Cellobiose	6	DM Glucose	Cellobiose	0.5	6.75	0
DM Fructose	4	DM Glucose	Fructose	0.5	6.75	0
DM Glycogen	6	DM Glucose	Glycogen	0.5	6.75	0
DM Lactose	4	DM Glucose	Lactose	0.5	6.75	0
DM Sucrose	6	DM Glucose	Sucrose	0.5	6.75	0

^a^ Number of resuscitated, biologically independent replicates that were used for the corresponding treatment

^b^ BAC, Benzalkonium Chloride

### Growth responses to extrinsic environmental stresses are consistent with previous literature

To represent isolates’ growth ability in the presence of osmotic, pH, and sanitizer stress conditions, isolates were classified by ability to grow (ΔOD_600_ ≥ 0.1) in all (Growth [G]), some (Variable [VAR]), or no (No Growth [NG]) replicates of each treatment (Table 2). To verify our treatment conditions could provide plausible tests of *L. monocytogenes* stress responses, we first analyzed results for isolates’ ability to grow and their growth rates as a whole, without separating by persistent or sporadic status.

**Table 2:** Number of the 95 *L. monocytogenes* isolates with a given growth status for each treatment condition

Treatments^a^	Growth	Variable	No Growth	*p*-value^b,c^
Stress Conditions (tested in nutritive media [BHI])				
Control	95	0	0	–
5% NaCl	95	0	0	No test
10% NaCl	51	44	0	**2.2 × 10**^**–16**^
pH of 5.2	90	5	0	0.059
pH of 9.2	90	5	0	0.059
BAC 2 μg/mL	0	95	0	**2.2 × 10**^**−16**^
BAC 5 μg/mL	0	49	46	**2.2 × 10**^**− 16**^
Energy Source Utilization (tested in chemically defined media [DM])				
DM Control	46	47	2	–
DM Cellobiose	60	35	0	0.052
DM Fructose	47	48	0	0.62
DM Glycogen	33	52	10	**0.021**
DM Lactose	1	11	83	**2.2 × 10**^**−16**^
DM Sucrose	1	18	76	**2.2 × 10**^**− 16**^

^a^ See Table 1 for formulations of each treatment

^b^ *p*-values, for the Fisher’s Exact test, for difference in growth response category between control and individual treatments in the same stress condition or energy source test category

^c^ Bolded *p*-values are statistically significant at *p* < 0.05

Isolates’ ability to grow was not significantly different from control BHI media for the 5% NaCl and pH 5.2 & 9.2 conditions. Isolates had significantly reduced ability to grow in 10% NaCl and 2 & 5 μg/mL BAC. BAC 5 μg/mL media was the least likely to support growth, with just above 50% (49/95) of isolates having variable growth and the rest of the panel not growing at all. While pH 5.2 and 9.2 trended to be significantly different (*p* = 0.052), there were only fives isolates that were in variable growth. Those five isolates with variable growth at pH 5.2 and pH 9.2 were not the same isolates across the two treatments.

Overall, stress conditions decrease growth rate among *L. monocytogenes* isolates that were able to grow (Additional file 1: Figure S1, overall analysis of variance [ANOVA] treatment effect *p* < 0.001). When comparing all extrinsic stress conditions, all treatment means were significantly lower than the control of normal Brain Heart Infusion (BHI) media (Tukey’s HSD, *p*-value = 0.05 threshold). The conditions of 5% NaCl, BAC 2 μg/mL, and pH 9.2 were all indistinguishable (Additional file 1: Figure S1), with remaining treatments showing even lower growth rates. Stress condition treatments were separated into three individual groups: salt, pH, and sanitizer. Within each group, growth rates are significantly different by dose (i.e. BAC 2 μg/mL results are significantly different from BAC 5 μg/mL, and so on). This expected dose-dependent effect was used as a confirmation that our treatment levels were reasonable.

As a species, *L. monocytogenes* is relatively resistant to many environmental stresses [48, 49]. It is not surprising that all isolates were always able to grow in 5 % salt, and all showed at least variable growth in 10 % salt, as *L. monocytogenes* is known to grow at high salt concentrations (up to 10% NaCl w/v) [24, 49]. Similarly, most isolates were always able to grow in both acidic (pH 5.2) and alkaline (pH 9.2) conditions, and it is known *L. monocytogenes* can survive and grow at low pHs (≥ 4.7) and high pHs (≤ 9.2) [23, 49]. As for the BAC data, treatment with 2 & 5 μg/mL allowed, at best, variable growth with significantly reduced growth rates. While industry uses a variety of different sanitizers, the concentration of BAC needed for complete inhibition of growth is around 60 μg/mL [50], a level that is justified, as this study showed that some, but not all, isolates are able to grow when exposed to lower concentrations. Overall, these data align with what has been seen already in literature for treatment effects of salt, pH, and sanitizer stress on growth of *L. monocytogenes* isolates, and this study adds substantial data on strain-to-strain variability. Other studies have focused on strain-to-strain variability and have found similarly variable results, not classifying strains as persistent or sporadic [51–54].

### Ability to utilize various energy sources in chemically defined media varies by isolate

*L. monocytogenes* was also examined for its ability to grow on various energy sources in chemically defined media (DM; see Table 2). The control condition data, DM Glucose, were split between consistent (*n* = 46) and variable (*n* = 47) growth with two isolates that never grew. Comparatively, DM Cellobiose maintained more consistent growth of isolates (*n* = 60) than any other treatment or the control. Only DM Cellobiose and Fructose conditions had zero No Growths; while DM Lactose and Sucrose had the most No Growths. DM Glycogen, Lactose, and Sucrose were the only treatments that had significantly different growth distributions than control DM Glucose (*p* < 0.05 for all), all with reduced ability to support growth. Cellobiose showed a trend towards supporting more growth than control (*p* = 0.052).

Growth rates of the *L. monocytogenes* isolates were not as varied in DM (Additional file 2: Figure S2). When comparing the treatments to the control (DM Glucose), only DM Lactose and Sucrose gave significant differences in growth rates (Tukey’s HSD test, p < 0.05) and overall was reduced, compared to control. These two treatments are also the same treatments that are least likely to support growth (Table 2). One important note, in these analyses, is that the definition of growth is a given change in optical density (OD) over time. This a created a growth rate limit of detection of ΔOD_600_ ≥ 0.1/24 h = 0.004ΔOD_600_/h; therefore, growth below this threshold was excluded.

### Defined media and supported growth

The results for which energy sources support the growth of *L. monocytogenes* are mostly consistent with previous studies in chemically DM, with this work testing a larger panel of energy sources and isolates. Most isolates were able to grow on glucose, cellobiose, fructose, and glycogen, whereas lactose and sucrose only rarely supported growth. The DM formula used in this study was a version of the formula used by Amezaga et al., the only difference was the use of different carbohydrates. Amezaga et al. stated that their media supported *L. monocytogenes* growth on glucose; however, no other carbohydrates were tested [55]; data in the study reported here suggests other growth factors may be required for robust growth of many strains for some carbohydrates.

A similar DM formula, developed by Premaratne et al., supported growth on fructose, cellobiose, and a few other energy sources not tested here, but not on lactose, sucrose, and other energy sources not tested here; glycogen data was not reported on in Premaratne et al. [56]. The major differences between these two DM formulae is that Amezaga et al. had added other materials like α-lipoic acid in ethanol and different phosphate salts [55]. While both DMs supported growth, only Premaratne et al. looked at multiple carbon sources besides glucose. The Premaratne formula used 10.0 g/L of glucose (equivalent to 55.5 mM) and did not specifically state the concentrations of the other tested sugars [56]. Thus, it is assumed that 10.0 g/L was used for all of the tested sugars. In contrast, all media in this study were formulated with 25 mM of a sole energy source. It is possible, though unlikely, that the relatively lower molar concentration of energy sources in this work could contribute to differences between each energy sources’ data.

The results presented in this study are consistent with other studies that show growth is supported by glucose, cellobiose and fructose [55–57], but the literature varies on if lactose and sucrose support *L. monocytogenes* growth [49], and glycogen has not been extensively studied [57]. In this study, isolates grew more consistently on cellobiose than on the control condition of glucose and showed a trend towards faster growth rates. The other DM formulation papers discussed above used glucose as their main energy source and reported consistent growth. Specifically, they reported consistent growth for three replicates of the common lab strain ATCC 23704 [55] and unknown replicates of strains Scott A, V7, CA, OH, ATCC 19115, and 28 unspecified dairy isolates [56]. Given that this study tested a larger panel of isolates these results suggest that cellobiose may be a better sole energy source for formulating DM to support the growth of a wide range of *L. monocytogenes* isolates from a deli-environment.

### Cellobiose

There are a few intriguing implications of the possibly increased ability of cellobiose to support growth over glucose. Since cellobiose is comprised of two glucose molecules, a dimer, one could assume growth on cellobiose should be similar to glucose. However, as there is a slight difference favoring cellobiose, there are at least three possible explanations for this difference. First, *L. monocytogenes* can be found in many different environments, but is common within the soil as a saprotroph [58]. As cellobiose is very common in soil, which is made up of decaying plant matter, and free glucose is rare, *L. monocytogenes* may have has adapted for comparatively better growth on cellobiose-rich substrates.

Second, it is possible that cellobiose is more energetically favorable compared to glucose metabolism. A few studies have found that in the presence of cellobiose, the main transcriptional activator of virulence genes, *prfA*, is down-regulated [57–60] – at least in part due to substrate-specific phosphotransferase system (PTS) importation [61] directly linked to virulence gene repression [62]. After PTS import of cellobiose, the substrate is phosphorylated, cleaved into glucose and glucose-6-P, and subsequently catabolized by the Embden-Meyerhof pathway [57, 63] like glucose. It is not clear which carbohydrate, glucose or cellobiose, would be more energetically favorable in DM based on the reduction of the metabolic burden of virulence gene expression and the cost of PTS transport.

Finally, the cellobiose treatment may have provided more gross energy simply due to formulating media on a mM basis. The implication of formulating our media on a mM basis is that there was an equal concentration of cellobiose and glucose molecules in each media. Since cellobiose is effectively broken down into two glucoses, it may be possible that cellobiose supported more growth because it effectively became twice as much available glucose, and from a single energetic importation.

### Glycogen

The DM data suggests that some deli-isolated *L. monocytogenes* can grow on media with glycogen as the primary energy source, which has not been previously reported by papers that developed chemically defined media. The total growth on glycogen is low, usually around a ΔOD_600_ of + 0.15. Still, according to Bergey’s manual of 2015, *L. monocytogenes* is known to not have any acid production from glycogen [49]. This discrepancy may be due to different methods to determine growth. The study presented here did not evaluate acid production from carbohydrate sources. Another caveat is that 10/95 isolates never grew in glycogen treated media, and all were from a single PFGE type, suggesting there may be sub-populations of these *L. monocytogenes* that differ in glycogen utilization.

### Lactose and sucrose

While the DM data suggests that most deli-associated *L. monocytogenes* isolates are unable to grow with lactose or sucrose as the sole energy source, there is intriguing variability in these phenotypes. Specifically, at least one isolate was able to consistently grow on each of these sugars, and a few more isolates showed variable growth. This variability of growth is particularly interesting for lactose, as *L. monocytogenes* can be isolated from dairy products [48], and unpasteurized dairy products have long been identified as high-risk foods for listeriosis [3]. However, the DM Lactose data suggests that deli-associated *L. monocytogenes* cannot grow well on lactose, by itself, in chemically defined media. It would be interesting to compare these results to growth of these same deli isolates to the growth of dairy-isolated *L. monocytogenes* in lactose-supplemented DM. In general, future work is needed studying strains of *L. monocytogenes*, from various sources, grown on multiple energy sources in different environments to assess if the variability is more a function of the strains, environments, or media components.

### Persistent and sporadic isolates from deli sources do not differ in extrinsic stress tolerance or energy sources utilization

**Fig. 1:**
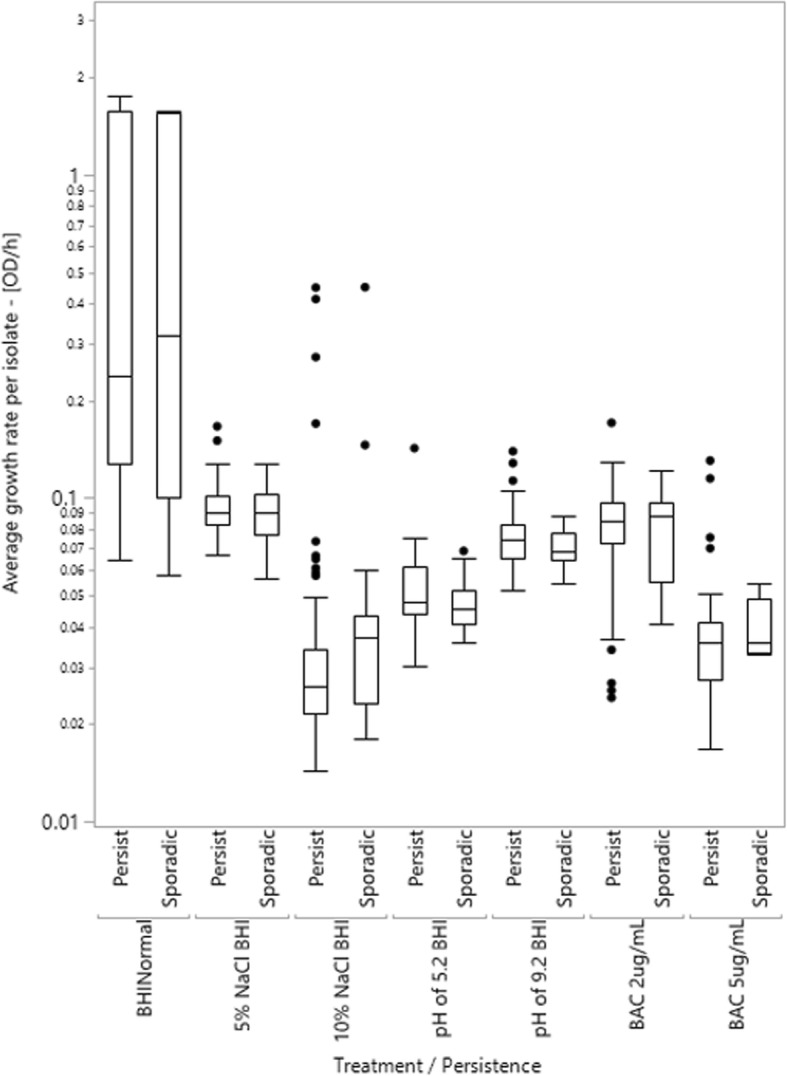
Box plots of average growth rate for *L. monocytogenes* isolates exposed to stress conditions, separated by their persistence factor. Data includes the average of only replicates where growth was observed (ΔOD_600_ ≥ 0.1), in log scale. The box represents the interquartile range (IQR), the line represents the median of the treatment, whiskers are drawn to the furthest point within 1.5 x IQR from the box, and the points are outliers of the data. No significant differences were observed in average growth rate between persistent and sporadic isolates for any treatment

**Fig. 2:**
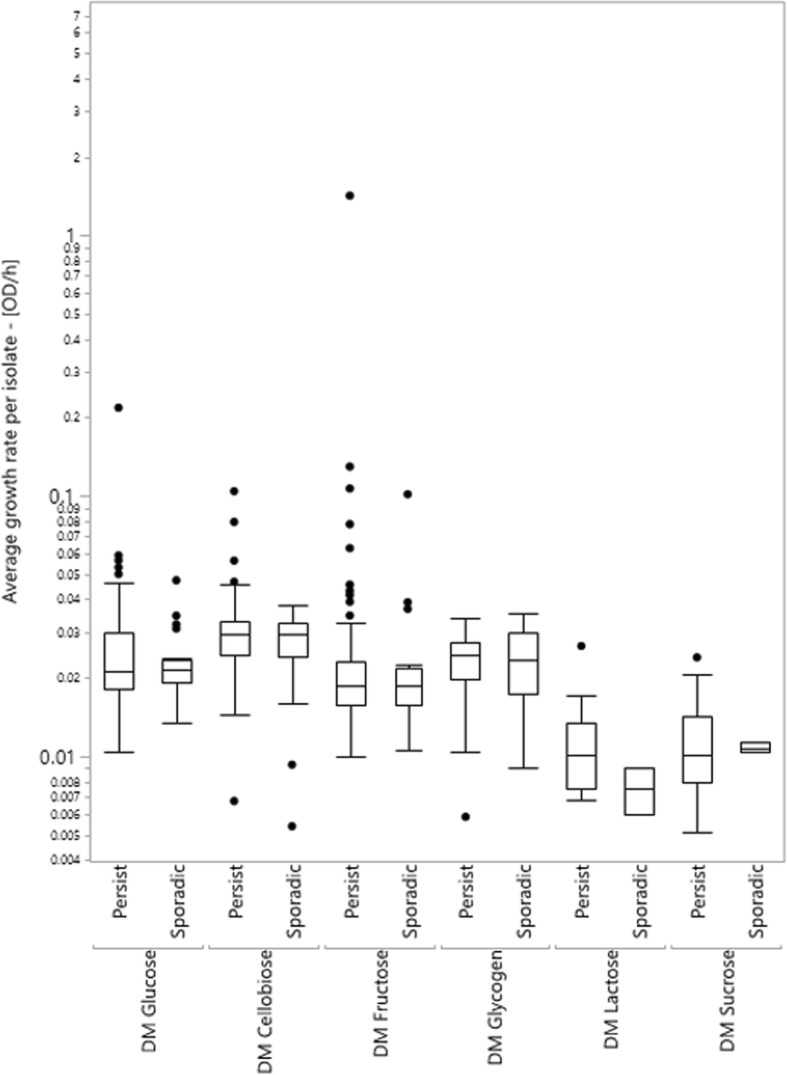
Box plots of average growth rate for *L. monocytogenes* isolates grown in chemically defined media (DM), separated by their persistence factor. Data only includes replicates whose growth was observed (ΔOD_600_ ≥ 0.1), in log scale. The box represents the interquartile range (IQR), the line represents the median of the treatment, whiskers are drawn to the furthest point within 1.5 x IQR from the box, and the points are outliers of the data. No significant differences were observed in average growth rate between persistent and sporadic isolates for any treatment

To test if persistent and sporadic isolates differ in relevant phenotypes the growth rate and ability to grow data were reanalyzed, separating isolates by persistence status (Figs. 1 and 2 are reanalyzed versions of Additional file 1: Figure S1 and Additional file 2: Figure S2, respectively). Mean growth rate did not differ systematically or statistically between isolates of persistent and sporadic strains for any treatments (*p* > 0.05 in all cases, by t-test). In addition, overall tests of data from extrinsic stress conditions and energy source usage were non-significant for the persistence factor (ANOVA, *p* = 0.82 & *p* = 0.22, respectively) and the interaction of persistence and treatment (ANOVA, *p* = 0.79 & *p* = 0.92, respectively). This suggests that there is no interaction effect between treatment and persistence of deli-associated *L. monocytogenes* on growth rate.

Isolates’ ability to grow was also reanalyzed to account for persistence status (Table 3). None of the treatments showed a significant difference in the proportion of isolates able to grow, or not grow, compared to control conditions for isolates of persistent or sporadic strains (*p* > 0.05 in all cases that were testable). From both of these assays, it is unlikely that isolates of persistent strains are able to grow better in extrinsic stress environments or on various energy sources than genetically similar isolates of sporadic strains.

**Table 3:** Number of the 95 *L. monocytogenes* isolates with a given growth status for each treatment condition, testing differences between persistent and sporadic isolates

Treatments^a^	Growth (%)^b^		Variable (%)		No Growth (%)		*p*-value^c^
	Persistent	Sporadic	Persistent	Sporadic	Persistent	Sporadic	
Stress Conditions in Nutritive Media (BHI)							
Control	74 (100)	21 (100)	0	0	0	0	1.0
5% NaCl BHI	74 (100)	21 (100)	0	0	0	0	1.0
10% NaCl BHI	41 (55)	10 (47)	33 (45)	11 (53)	0	0	0.62
pH of 5.2	69 (93)	21 (100)	5 (7)	0	0	0	0.58
pH of 9.2	70 (95)	20 (95)	4 (5)	1 (5)	0	0	1.0
BAC 2 μg/mL	0	0	74 (100)	21 (100)	0	0	1.0
BAC 5 μg/mL	0	0	41 (55)	8 (38)	33 (45)	13 (62)	0.22
Energy Source Utilization (tested in chemically defined media [DM])							
DM Glucose	38 (51)	8 (38)	34 (46)	13 (62)	2 (3)	0	0.46
DM Cellobiose	50 (68)	10 (48)	24 (32)	11 (52)	0	0	0.12
DM Fructose	38 (51)	9 (43)	36 (49)	12 (57)	0	0	0.62
DM Glycogen	24 (32)	9 (43)	41 (55)	11 (52)	9 (12)	1 (5)	0.55
DM Lactose	1 (1)	0	9 (12)	2 (10)	64 (87)	19 (90)	1.0
DM Sucrose	1 (1)	0	15 (20)	3 (14)	58 (78)	18 (86)	0.81

^a^ See Table 1 for treatment formulations

^b^ Numbers in parentheses are the percentage of the total persistent or total sporadic isolates

^c^ *p*-values were determined by Chi Square Test, or Fisher’s exact test if any cells contained less than five isolates

To check for robustness of these conclusions, phylogenetic clades established in previous research ([32] and Additional file 3: Table S1) were added as a factor in the model for persistence and treatment effects on growth rates and ability to grow. Phylogenetic clade did not have a significant effect in the model for grow rate for either stress tolerance (*p* = 0.1) or energy source utilization (*p* = 0.5). While clade did have a significant effect in the model of ability to grow for both stress tolerance (*p* = 0.03) and energy source utilization (*p* = 0.01), these models gave unstable estimates for the other parameters. Importantly, in all four models, the persistence-treatment interaction and the main effect of persistence were always, still, not significant (*p*-values > 0.6 in all cases). Further, the main effect of treatment was always significant (*p* < 0.001 in all cases). Therefore, we can conclude clade does not meaningfully impact the conclusion that isolates of persistent strains are able to grow better in extrinsic stress environments or on energy sources than otherwise similar isolates of sporadic strains that are also from retail delis.

### Discussion of the differences between persistent and sporadic *L. monocytogenes* isolates in their growth rates and their ability to grow

Previous reports have shown persistent and non-persistent *L. monocytogenes* strains and how they react to varying salt concentrations, acidity conditions, and quaternary ammonium compound (QAC) concentrations [12, 28, 40, 46, 64]. Recently, a report by Magalhães et al. looked at BAC resistance, osmotic pressure, and pH conditions on growth kinetics, in 41 persistent and non-persistent strains from three cheese processing plants classified by PFGE subtyping. They concluded that there were not significant differences in lag time or growth rate between persistent and non-persistent strains in 50 μg/mL of BAC. The BAC data presented in this study is consistent with the sanitizer data section of Magalhães et al.’s report [28]. As for the other two stresses, the osmotic pressure and acid data presented here is in conflict with Magalhães et al.’s data [28]. While they were able to provide evidence that lag time was shorter for persistent strains at 2.5, 4, 8% NaCl and pH 5, there were no significant differences in the data presented here between persistent and sporadic strains grown under similar conditions. For the interaction of persistence and acid tolerance, Lundén et al., that showed 17 persistent strains, from three meat-processing plants, had higher tolerance, less log reduction, to acidic (pH 2.4 for 2 h) conditions than 23 non-persistent strains [12]. In that study, growth under sub-lethal acid stress was not tested.

There could be at least two possible contributions to the differing results of persistent strain growth under extrinsic stresses: classification methods and small sample sizes. The Lundén study identified 34 different PFGE subtypes, of which 12 were persistent and 22 were non-persistent. This means that Lundén et al.’s comparisons of persistent and non-persistent strains used a restriction fragment pattern analysis method known to be influenced by mobile elements such as prophage [12]. In addition, to empirically identify persistence, they only classified strains persistent if they were isolated 5 or more times in a span of 3 months (in comparison, this study used a WGS-based SNP-difference metric). Truly persistent strains isolated less frequently might have been classified as non-persistent, as a logical consequence of the empirical rules defined within the research.

Comparatively, other studies have relatively smaller sample sizes (*n* < 50) of isolates [12, 28, 30, 65–67] compared to the 95 isolates analyzed here. To overcome the limitations of previous subtyping methods and smaller samples sizes, the study presented here used persistent strains identified by WGS SNP-based analyses that can account for certain prophage effects [32]. We included 74 persistent strains, representing 20 putative persistence events, and 21 closely related sporadic strains of *L. monocytogenes* from retail delis, providing increased statistical power. Within the full data set, it does not seem that isolates of persistent strains of *L. monocytogenes* grow faster or have a better ability to grow than sporadic strains. It seems that, more likely, isolates of persistent strains rely on permissive environmental conditions rather than phenotypic adaptations.

Finally, all of the isolates used in this study were isolated from retail delis. While this lack of diversity is a limitation to the study’s generalizability, the narrow focus of the project was necessary to provide a valid, if limited, test of growth for retail deli-isolated strains of *L. monocytogenes* at 37 °C, responding to various environmental and energy source factors. Future studies could explore phenotypic differences between persistent and sporadic strains (i) including other extrinsic or intrinsic factors, such as colder temperatures, 4–10 °C and 20–25 °C, and in solid media and (ii) isolates from multiple locations, such as clinical and natural environments.

### Conclusion for persistence and growth rates and ability to grow of *L. monocytogenes*

This study set out to characterize different phenotypic responses of isolates from persistent and sporadic strains. Extrinsic stress conditions and various carbohydrate sources have significant effects on *L. monocytogenes*’ ability to grow and growth rate. However, when comparing growth between isolates of persistent and sporadic strains from retail delis at 37 °C, there does not seem to be any differences in the ability to grow or growth rates. These results indicate that *L. monocytogenes* isolates of persistent strains are likely not persistent due to strain specific phenotypes in the extrinsic factors tested here (salt, pH, sanitizer, and energy source stress; acknowledging that lower temperatures, water activity, non-carbohydrate nutrients, etc., were not studied here). Rather, persistence is likely a combination of environmental conditions and factors. From this conclusion, the authors believe future research on the control of persistent *L. monocytogenes* would be better focused on improving environmental-based monitoring and seek-and-destroy strategies [13] to eliminate harborage sites, which are known to contribute to persistence. Other work could be to investigate differences between persistent and sporadic *L. monocytogenes* in refrigerated, solid media from multiple source environments. This work also adds to literature on *Listeria* metabolism by finding evidence for strain-to-strain variability of *L. monocytogenes*’ energy source utilization, particularly with glycogen, lactose, and sucrose.

## Materials and methods

### Strain selection

In this study, 95 isolates (74 [77%] persistent and 21 [23%] sporadic) that represent 20 putative persistence events and 21 sporadic strains were analyzed. These isolates were included in a previous study that used WGS based phylogenetics to identify persistent strains from retail delis [8, 32]. These delis were locations within larger retail grocery establishments that sell a variety of processed meats sliced on site. *L. monocytogenes* isolates were identified by consistently swabbing 28 food contact and nonfood contact locations in the delis; additional details on the sampling project are reported in [8]. Stasiewicz et al. applied WGS SNP-based phylogenetics to the strains and argued that certain well-supported clades identified putative persistence events were unique to a single deli, unique to a single state, or spanned multiple states [32]. These isolates were identified as representing putative persistent strains. Critical metadata for all isolates selected for sequencing are found in Additional file 3: Table S1 and additional metadata are stored in the database www.foodmicrobetracker.com.

While the referenced study [32] analyzed 175 isolates, the authors selected 95 isolates of those 175 as this number is appropriate for high-throughput analysis in microtiter plates. The 95 chosen isolates were systematically selected. First, only strains that were associated with statistically-significant WGS SNP evidence for persistence were selected [32]. Second, the panel included all sporadic isolates closely related to the persistent strains (specifically, were in the same clade). Third, only isolates physically available from Dr. Oliver’s lab at Purdue University were acquired. This consolidated 175 isolates to 105 candidate isolates for the phenotyping panel. Of the 105 candidates, some putative persistence events were more represented than others were, so 10 randomly selected isolates were discarded from events that already had sufficient representation. Overall, 95 total isolates, 74 persistent and 21 sporadic, were selected and represent 20 putative persistence events and 21 closely related sporadic strains. This imbalance of persistent and sporadic strains was a consequence of the relative rarity of sporadic isolates in the original sampling study. This should not impact the power of the study to detect differences between persistent and sporadic strains overall, but may lower the power of analyses by clade.

Isolates were maintained at − 80 °C in 12.5% v/v glycerol-brain-heart infusion (BHI) media in 96-well microplate format (Corning Clear Polystyrene 96-Well Microplates 360 μL, Corning, Tewksbury, MA). Before being assayed in the Bioscreen C, isolates were resuscitated from frozen stocks by pre-growth in control media (BHI or DM Glucose, described below) at 37 °C, for optimal growth, for 18 to 24 h (Overnight, O/N) with shaking at 150 rpm, again in the 96-well microplates.

### Treatment media

There were multiple treatment media used in this project (Table 1). This project used nutritive media to create stress conditions and chemically defined media to assay growth in different energy sources. The control media were either BHI (Sigma-Aldrich, St. Louis, MO) or a chemically DM, specifically formulated for *Listeria* species [55], which uses glucose as a control energy source. As the tested *L. monocytogenes* strains come from retail delis, three different extrinsic stresses were tested, that are commonly associated with persistence – osmotic pressure, acidic and alkali pH, and a sanitizer. Therefore, the following media were made: BHI with 5% or 10% w/v NaCl; BHI at pH 5.2 or 9.2 BHI (adjusted with 3 M HCl or 3 M NaOH); BHI with 2 or 5 μg/mL benzalkonium chloride (BAC), a QAC and common industry sanitizer [33]. Specifically, different environments can harbor various amounts of salt, acid and alkali, and sanitizer [28, 29, 68, 69]. The concentrations listed here are the final concentrations used in the test media (i.e. 5.5% NaCl w/v was created so that a 1:10 dilution would have a 5.0% NaCl w/v final concentration).

Energy sources were also assayed, such as glucose (control), cellobiose, fructose, glycogen, lactose, and sucrose. DM was used to focus in on the growth rate and ability to grow given different carbohydrate sources. The DM energy sources were substituted at the same initial concentration (25 mM) as directed in previous literature [55]. Each energy source was chosen to represent a source the pathogen may encounter within a food environment. Cellobiose was for observing *L. monocytogenes*’ ability to grow on plant matter (vegetables). Fructose was representative of fruit sugars (fruits). Glycogen was representative of muscle tissue (meats). Lactose was representative of milk sugars (dairy products). Sucrose was representative of refined sugar (sweets).

### Growth assay

O/N cultures were transferred from the resuscitated 96-well plate to a 100-well Honeycomb Bioscreen Plate (Growth Curves USA, Piscataway Township, NJ) in the treatment specific media (20 μL O/N culture with 180 μL of fresh, treatment media, i.e. a 10-fold dilution). A 10-fold dilution inoculation was chosen so that the initial inoculum was above the machine’s detection limit, and therefore initial density and lag phase could be obtained at a time point zero. Cultures were then grown for 24 h, at 37 °C, in the Bioscreen C (Growth Curves USA, Piscataway Township, NJ) Automated Growth Curve Analysis System. The Bioscreener software recorded the OD_600_ of each of the 100 wells from time zero to 24 h later in 15 min intervals, with shaking at medium-intensity 15 s before each interval reading. This data collection scheme allowed for capture of the starting inoculation levels, final growth level, lag phase, and growth rate, as described below. Cultures were assayed for 3–6 biological replicates of each treatment by individual resuscitations from frozen stock cultures.

The specific growth temperature of 37 °C was utilized in the initial screen for relevant phenotypes because this temperature was experimentally convenient compared to refrigerated temperature work. Originally, the design of the experiment was for a high-throughput screen at 37 °C and to perform follow-up experiments at 4 °C, if there were observed significant differences, to gather data even more relevant to environmental survival. As there were no significant differences observed between growth of isolates of persistent and sporadic strains, at 37 °C, the 4 °C work was not attempted in this study.

Growth data was analyzed using an open-source regression tool specifically adapted to fitting food microbiological growth models to OD data [70]. The tool fits a Baranyi Roberts growth curve to the OD_600_ data. Curves were only fit to data where growth was observed, which is defined as ΔOD_600_ ≥ 0.1. Outputs would include the initial and final OD_600_, lag time, maximum exponential growth rate, doubling time, and ΔOD_600_ of calculated from each well. The initial analysis included isolates’ lag time, ΔOD, and growth rate. However, only growth rate analyses are presented, as the lag time was inversely related to growth rate and ΔOD was directly proportional to growth rate. Each isolate was grown at a minimum of three times and a maximum of six times. For each treatment, the growth rates were averaged for the control treatments. Initial OD_600_ readings for all wells of BHINormal and DM Glucose had means of 0.195 and 0.112 with standard deviations of 0.062 and 0.021, respectively, suggesting these isolates were inoculated to similar initial densities within their respective media.

### Data analysis

Growth parameter data was analyzed to compare both if isolates were able to grow and growth rate, if growth was observed. As for the isolates’ ability to grow, isolates were given the designations of “Growth,” (G) “Variable,” (VAR) or “No Growth” (NG) if they either grew in (ΔOD_600_ ≥ 0.1) every replicate of a treatment, grew in at least one replicate but not all, or did not grow in any of the replicates of a treatment, respectively. Significant differences were tested in the number of isolates for each growth category for each treatment compared to its respective control (Control [BHINormal] and DM Control [DM Glucose], for stress response and energy source utilization, respectively) using χ^2^ tests (or Fisher’s Exact tests if any cell had < 5 counts). When persistence was examined for its effect on growth, comparisons to a control were not used. Fisher’s Exact tests were utilized for singular treatments split by persistent and sporadic connotations.

To analyze growth rate data, data was aggregated across replicates by calculating the mean lag time, max growth rate, and ΔOD, for each strain for each treatment where growth was observed (ΔOD_600_ ≥ 0.1). Then, the data was tested for the effects of treatment, persistence, and the interaction of treatment*persistence on growth parameters using Analysis of Variance (ANOVA). Plotting and further statistical analyses were performed in JMP (JMP Pro 13.0.0, SAS Inc., Cary, NC). Phylogenetic clades were also examined for their effect as a main effect into the previously described models for both growth rates and ability to grow.

## Supplementary information

**Additional file 1: Figure S1.** Box plots of the average growth rate for *L. monocytogenes* isolates exposed to stress conditions. Data includes the average of only replicates whose growth was observed (ΔOD_600_ ≥ 0.1), in log scale. The box represents the interquartile range (IQR), the line represents the median of the treatment, whiskers are drawn to the furthest point within 1.5 x IQR from the box, and the points are outliers of the data. Groups were created by use of Tukey’s HSD, where the same letters indicate means that are not different from each other.

**Additional file 2: Figure S2.** Box plots of average growth rate for *L. monocytogenes* isolates grown in chemically defined media (DM) at 25 mM concentrations of each energy source. Data includes averages of only replicates whose growth was observed (ΔOD_600_ ≥ 0.1), in log scale. Groups were created by use of Tukey’s HSD, where the same letters indicate means that are not different from each other.

**Additional file 3: Table S1.** Metadata of the 95 *L. monocytogenes* isolates analyzed.

## Data Availability

The datasets generated and analyzed are available from the corresponding author on reasonable request.

## Abbreviations

ANOVA: Analysis of Variance
BAC: Benzalkonium chloride
BHI: Brain Heart Infusion
DM: Defined media
G: Growth
IQR: Interquartile range
MLST: Multilocus Sequence Typing
NG: No Growth
O/N: Overnight
OD: Optical density
PFGE: Pulse field gel electrophoresis
PTS: Phosphotransferase system
QAC: Quaternary ammonium compound
RTE: Ready-to-eat
SNP: Single nucleotide polymorphism
VAR: Variable
WGS: Whole genome sequencing

## References

[1] Scallan E, Hoekstra RM, Angulo FJ, Tauxe RV, Widdowson MA, Roy SL (2011). Foodborne illness acquired in the United States--major pathogens. Emerg Infect Dis.

[2] Müller A, Rychli K, Muhterem-Uyar M, Zaiser A, Stessl B, Guinane CM (2013). Tn*6188* - a novel transposon in *Listeria monocytogenes* responsible for tolerance to benzalkonium chloride. PLoS One.

[3] CFSAN (2003). Quantitative assessment of the relative risk to public health from foodborne *Listeria monocytogenes* among selected categories of ready-to-eat foods.

[4] Luchansky JB, Chen Y, Porto-Fett ACS, Pouillot R, Shoyer BA, Johnson-DeRycke R (2017). Survey for *Listeria monocytogenes* in and on ready-to-eat foods from retail establishments in the United States (2010 through 2013): assessing potential changes of pathogen prevalence and levels in a decade. J Food Prot.

[5] Smith A, Moorhouse E, Monaghan J, Taylor C, Singleton I (2018). Sources and survival of *Listeria monocytogenes* on fresh, leafy produce. J Appl Microbiol.

[6] Vongkamjan K, Fuangpaiboon J, Turner MP, Vuddhakul V (2016). Various ready-to-eat products from retail stores linked to occurrence of diverse *Listeria monocytogenes* and *Listeria* spp. isolates. J Food Prot.

[7] Zhu Qi, Gooneratne Ravi, Hussain Malik (2017). Listeria monocytogenes in Fresh Produce: Outbreaks, Prevalence and Contamination Levels. Foods.

[8] Simmons C, Stasiewicz MJ, Wright E, Warchocki S, Roof S, Kause JR (2014). *Listeria monocytogenes* and *Listeria* spp. contamination patterns in retail delicatessen establishments in three U.S. states. Food Prot..

[9] Pouillot R, Gallagher D, Tang J, Hoelzer K, Kause J, Dennis SB (2015). *Listeria monocytogenes* in retail delicatessens: an interagency risk assessment-model and baseline results. J Food Prot.

[10] Currie A, Farber JM, Nadon C, Sharma D, Whitfield Y, Gaulin C (2015). Multi-province listeriosis outbreak linked to contaminated deli meat consumed primarily in institutional settings, Canada, 2008. Foodborne Pathog Dis.

[11] Brown LG, Hoover ER, Ripley D, Matis B, Nicholas D, Hedeen N (2016). Retail deli slicer cleaning frequency-- six selected sites, United States, 2012. MMWR Morb Mortal Wkly Rep.

[12] Lundén J, Tolvanen R, Korkeala H (2008). Acid and heat tolerance of persistent and nonpersistent *Listeria monocytogenes* food plant strains. Lett Appl Microbiol.

[13] Malley TJ, Butts J, Wiedmann M (2015). Seek and destroy process: *Listeria monocytogenes* process controls in the ready-to-eat meat and poultry industry. Food Prot..

[14] Etter Andrea J., Hammons Susan R., Roof Sherry, Simmons Courtenay, Wu Tongyu, Cook Peter W., Katubig Alex, Stasiewicz Matthew J., Wright Emily, Warchocki Steven, Hollingworth Jill, Thesmar Hilary S., Ibrahim Salam A., Wiedmann Martin, Oliver Haley F. (2017). Enhanced Sanitation Standard Operating Procedures Have Limited Impact onListeria monocytogenesPrevalence in Retail Delis. Journal of Food Protection.

[15] Bolocan AS, Pennone V, O'Connor PM, Coffey A, Nicolau AI, McAuliffe O (2017). Inhibition of *Listeria monocytogenes* biofilms by bacteriocin-producing bacteria isolated from mushroom substrate. J Appl Microbiol.

[16] da Silva EP, De Martinis EC (2013). Current knowledge and perspectives on biofilm formation: the case of *Listeria monocytogenes*. Appl Microbiol Biotechnol.

[17] Wang J, Ray AJ, Hammons SR, Oliver HF (2015). Persistent and transient *Listeria monocytogenes* strains from retail deli environments vary in their ability to adhere and form biofilms and rarely have *inlA* premature stop codons. Foodborne Pathog Dis.

[18] Habimana O, Meyrand M, Meylheuc T, Kulakauskas S, Briandet R (2009). Genetic features of resident biofilms determine attachment of *Listeria monocytogenes*. Appl Environ Microbiol.

[19] Jeong DK, Frank JF (1994). Growth of *Listeria monocytogenes* at 10 degrees C in biofilms with microorganisms isolated from meat and dairy processing environments. J Food Prot.

[20] Fox EM, Solomon K, Moore JE, Wall PG, Fanning S (2014). Phylogenetic profiles of in-house microflora in drains at a food production facility: comparison and biocontrol implications of *Listeria*-positive and -negative bacterial populations. Appl Environ Microbiol.

[21] Martinez-Suarez JV, Ortiz S, Lopez-Alonso V (2016). Potential impact of the resistance to quaternary ammonium disinfectants on the persistence of *Listeria monocytogenes* in food processing environments. Front Microbiol.

[22] Nakamura H, Takakura K, Sone Y, Itano Y, Nishikawa Y (2013). Biofilm formation and resistance to benzalkonium chloride in *Listeria monocytogenes* isolated from a fish processing plant. Food Prot..

[23] Ferreira V, Wiedmann M, Teixeira P, Stasiewicz MJ (2014). *Listeria monocytogenes* persistence in food-associated environments: epidemiology, strain characteristics, and implications for public health. Food Prot.

[24] Mcclure PJ, Roberts TA, Oguru PO (1989). Comparison of the effects of sodium chloride, pH and temperature on the growth of *Listeria monocytogenes* on gradient plates and in liquid medium. Lett Appl Microbiol.

[25] Gerald McDonnell ADR (1999). Antiseptics and disinfectants: activity, action, and resistance. Clin Microbiol Rev.

[26] Stanga M. Sanitation: cleaning and disinfection in the food industry. Wiley-VCH, Weinheim: Germany; 2010. 611.

[27] Wilson A, Gray J, Chandry PS, Fox EM. Phenotypic and genotypic analysis of antimicrobial resistance among *Listeria monocytogenes* isolated from Australian food production chains. Genes (Basel). 2018;9(2):80. 10.3390/genes9020080 PMC5852576 29425131

[28] Magalhães R, Ferreira V, Brandao TR, Palencia RC, Almeida G, Teixeira P (2016). Persistent and non-persistent strains of *Listeria monocytogenes*: a focus on growth kinetics under different temperature, salt, and pH conditions and their sensitivity to sanitizers. Food Microbiol.

[29] Rychli K, Grunert T, Ciolacu L, Zaiser A, Razzazi-Fazeli E, Schmitz-Esser S (2016). Exoproteome analysis reveals higher abundance of proteins linked to alkaline stress in persistent *Listeria monocytogenes* strains. Int J Food Microbiol.

[30] Cherifi T, Carrillo C, Lambert D, Miniai I, Quessy S, Lariviere-Gauthier G (2018). Genomic characterization of *Listeria monocytogenes* isolates reveals that their persistence in a pig slaughterhouse is linked to the presence of benzalkonium chloride resistance genes. BMC Microbiol.

[31] Keto-Timonen R, Tolvanen R, Lunden J, Korkeala H (2007). An 8-year surveillance of the diversity and persistence of *Listeria monocytogenes* in a chilled food processing plant analyzed by amplified fragment length polymorphism. Food Prot..

[32] Stasiewicz MJ, Oliver HF, Wiedmann M, den Bakker HC (2015). Whole-genome sequencing allows for improved identification of persistent *Listeria monocytogenes* in food-associated environments. Appl Environ Microbiol.

[33] Moretro T, Schirmer BC, Heir E, Fagerlund A, Hjemli P, Langsrud S (2017). Tolerance to quaternary ammonium compound disinfectants may enhance growth of *Listeria monocytogenes* in the food industry. Int J Food Microbiol.

[34] da Silva FM, Kabuki DY, Kuaye AY (2015). Behavior of *Listeria monocytogenes* in a multi-species biofilm with *Enterococcus faecalis* and *Enterococcus faecium* and control through sanitation procedures. Int J Food Microbiol.

[35] Giaouris E, Chorianopoulos N, Doulgeraki A, Nychas GJ (2013). Co-culture with *Listeria monocytogenes* within a dual-species biofilm community strongly increases resistance of *Pseudomonas putida* to benzalkonium chloride. PLoS One.

[36] Kostaki M, Chorianopoulos N, Braxou E, Nychas GJ, Giaouris E (2012). Differential biofilm formation and chemical disinfection resistance of sessile cells of *Listeria monocytogenes* strains under monospecies and dual-species (with *Salmonella enterica*) conditions. Appl Environ Microbiol.

[37] van der Veen S, Abee T (2011). Mixed species biofilms of *Listeria monocytogenes* and *Lactobacillus plantarum* show enhanced resistance to benzalkonium chloride and peracetic acid. Int J Food Microbiol.

[38] Komora N, Bruschi C, Magalhães R, Ferreira V, Teixeira P (2017). Survival of *Listeria monocytogenes* with different antibiotic resistance patterns to food-associated stresses. Int J Food Microbiol.

[39] Allen KJ, Wałecka-Zacharska E, Chen JC, Katarzyna K-P, Devlieghere F, Van Meervenne E (2016). *Listeria monocytogenes* – an examination of food chain factors potentially contributing to antimicrobial resistance. Food Microbiol.

[40] Müller A, Rychli K, Zaiser A, Wieser C, Wagner M, Schmitz-Esser S (2014). The *Listeria monocytogenes* transposon Tn*6188* provides increased tolerance to various quaternary ammonium compounds and ethidium bromide. FEMS Microbiol Lett.

[41] Minarovicova J, Veghova A, Mikulasova M, Chovanova R, Soltys K, Drahovska H (2018). Benzalkonium chloride tolerance of *Listeria monocytogenes* strains isolated from a meat processing facility is related to presence of plasmid-borne *bcr*ABC cassette. Antonie Van Leeuwenhoek.

[42] Yu T, Jiang X, Zhang Y, Ji S, Gao W, Shi L (2018). Effect of benzalkonium chloride adaptation on sensitivity to antimicrobial agents and tolerance to environmental stresses in *Listeria monocytogenes*. Front Microbiol.

[43] Ortiz S, Lopez-Alonso V, Rodriguez P, Martinez-Suarez JV (2015). The connection between persistent, disinfectant-resistant *Listeria monocytogenes* strains from two geographically separate Iberian pork processing plants: evidence from comparative genome analysis. Appl Environ Microbiol.

[44] Laksanalamai P, Joseph LA, Silk BJ, Burall LS, Tarr CL, Gerner-Smidt P (2012). Genomic characterization of *Listeria monocytogenes* strains involved in a multistate listeriosis outbreak associated with cantaloupe in US. PLoS One.

[45] Luo L, Zhang Z, Wang H, Wang P, Lan R, Deng J (2017). A 12-month longitudinal study of *Listeria monocytogenes* contamination and persistence in pork retail markets in China. Food Control.

[46] Moura A, Criscuolo A, Pouseele H, Maury MM, Leclercq A, Tarr C (2016). Whole genome-based population biology and epidemiological surveillance of *Listeria monocytogenes*. Nat Microbiol.

[47] Jadhav S, Bhave M, Palombo EA (2012). Methods used for the detection and subtyping of *Listeria monocytogenes*. J Microbiol Methods.

[48] Buchanan RL, Gorris LGM, Hayman MM, Jackson TC, Whiting RC (2017). A review of *Listeria monocytogenes*: an update on outbreaks, virulence, dose-response, ecology, and risk assessments. Food Control.

[49] McLauchlin JR, Rees CE. *Listeria*. Bergey's Manual of Systematics of Archaea and Bacteria 2015:1–29.

[50] Kremer PH, Lees JA, Koopmans MM, Ferwerda B, Arends AW, Feller MM (2016). Benzalkonium tolerance genes and outcome in *Listeria monocytogenes* meningitis. Clin Microbiol Infect.

[51] Horlbog JA, Kent D, Stephan R, Guldimann C (2018). Surviving host - and food relevant stresses: phenotype of *L. monocytogenes* strains isolated from food and clinical sources. Sci Rep.

[52] Pouillot R, Hoelzer K, Chen Y, Dennis SB (2015). *Listeria monocytogenes* dose response revisited--incorporating adjustments for variability in strain virulence and host susceptibility. Risk Anal.

[53] Ciolacu L, Nicolau AI, Wagner M, Rychli K (2015). *Listeria monocytogenes* isolated from food samples from a Romanian black market show distinct virulence profiles. Int J Food Microbiol.

[54] Tang S, Stasiewicz MJ, Wiedmann M, Boor KJ, Bergholz TM (2013). Efficacy of different antimicrobials on inhibition of *Listeria monocytogenes* growth in laboratory medium and on cold-smoked salmon. Int J Food Microbiol.

[55] Amezaga MR, Davidson I, McLaggan D, Verheul A, Abee T, Booth IR (1995). The role of peptide metabolism in the growth of *Listeria monocytogenes* ATCC 23074 at high osmolarity. Microbiol..

[56] Premaratne RJ, Lin WJ, Johnson EA (1991). Development of an improved chemically defined minimal medium for *Listeria monocytogenes*. Appl Environ Microbiol.

[57] Slaghuis J, Joseph B, Goebel W, Goldfine H, Shen H (2007). Metabolism and physiology of *Listeria monocytogenes*. *Listeria monocytogenes*: pathogenesis and host response.

[58] Kentache T, Milohanic E, Cao TN, Mokhtari A, Ake FM, Ma Pham QM (2016). Transport and catabolism of pentitols by *Listeria monocytogenes*. J Mol Microbiol Biotechnol.

[59] Marr AK, Joseph B, Mertins S, Ecke R, Muller-Altrock S, Goebel W (2006). Overexpression of PrfA leads to growth inhibition of *Listeria monocytogenes* in glucose-containing culture media by interfering with glucose uptake. J Bacteriol.

[60] Stoll R, Mertins S, Joseph B, Muller-Altrock S, Goebel W (2008). Modulation of PrfA activity in *Listeria monocytogenes* upon growth in different culture media. Microbiol..

[61] Stoll R, Goebel W (2010). The major PEP-phosphotransferase systems (PTSs) for glucose, mannose and cellobiose of *Listeria monocytogenes*, and their significance for extra- and intracellular growth. Microbiol..

[62] Cao TN, Joyet P, Ake FMD, Milohanic E, Deutscher J. Studies of the *Listeria monocytogenes* cellobiose transport components and their impact on virulence gene repression. J Mol Microbiol Biotechnol. 2019:1–17. 10.1159/000500090 31269503

[63] Deutscher J, Moussan Désirée Aké F, Zebre A, Nguyen Cao T, Kentache T, Mai Ma Pham Q, et al. Carbohydrate utilization by *Listeria monocytogenes* and its influence on virulence gene expression. In: Hambrick EC, editor. *Listeria monocytogenes*: Food Sources, Prevalence and Management Strategies. New York: Nova science publishers; 2014. p. 49–76.

[64] Mereghetti L, Quentin R, Marquet-Van Der Mee N, Audurier A (2000). Low sensitivity of *Listeria monocytogenes* to quaternary ammonium compounds. Appl Environ Microbiol.

[65] Fox EM, Leonard N, Jordan K (2011). Physiological and transcriptional characterization of persistent and nonpersistent *Listeria monocytogenes* isolates. Appl Environ Microbiol.

[66] Mazza R, Mazzette R, McAuliffe O, Jordan K, Fox EM (2015). Differential gene expression of three gene targets among persistent and nonpersistent *Listeria monocytogenes* strains in the presence or absence of benzethonium chloride. Food Prot..

[67] Lim SY, Yap KP, Thong KL (2016). Comparative genomics analyses revealed two virulent *Listeria monocytogenes* strains isolated from ready-to-eat food. Gut Pathog.

[68] Aarnisalo K, Lundén J, Korkeala H, Wirtanen G (2007). Susceptibility of *Listeria monocytogenes* strains to disinfectants and chlorinated alkaline cleaners at cold temperatures. LWT - Food Sci Technol.

[69] Kang J, Stasiewicz MJ, Murray D, Boor KJ, Wiedmann M, Bergholz TM (2014). Optimization of combinations of bactericidal and bacteriostatic treatments to control *Listeria monocytogenes* on cold-smoked salmon. Int J Food Microbiol.

[70] Hoeflinger JL, Hoeflinger DE, Miller MJ (2017). A dynamic regression analysis tool for quantitative assessment of bacterial growth written in python. J Microbiol Methods.

