# Supplementary material for: Persistent and sporadic Listeria monocytogenes strains do not differ when growing at 37 °C, in planktonic state, under different food associated stresses or energy sources
Source: BMC Microbiol. 2019 Nov 19;19:257. doi: 10.1186/s12866-019-1631-3 (PMC6862832; doi:10.1186/s12866-019-1631-3)
Supplement: Supplementary file 1 — Additional file 1: Figure S1. Box plots of the average growth rate for L. monocytogenes isolates exposed to stress conditions. Data includes the average of only replicates whose growth was observed (ΔOD600 ≥ 0.1), in log scale. The box represents the interquartile range (IQR), the line represents the median of the treatment, whiskers are drawn to the furthest point within 1.5 x IQR from the box, and the points are outliers of the data. Groups were created by use of Tukey’s HSD, where the same letters indicate means that are not different from each other. [file 12866_2019_1631_MOESM1_ESM.docx]

## Supplemental Information


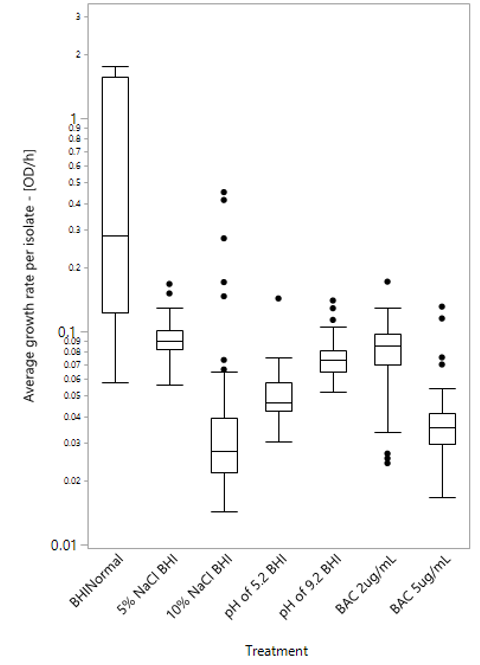


CD

C

A

B

D

B

B

**Figure S1**: Box plots of the average growth rate for *L. monocytogenes* isolates exposed to stress conditions. Data includes the average of only replicates whose growth was observed (ΔOD_600_ ≥0.1), in log scale. The box represents the interquartile range (IQR), the line represents the median of the treatment, whiskers are drawn to the furthest point within 1.5 x IQR from the box, and the points are outliers of the data. Groups were created by use of Tukey’s HSD, where the same letters indicate means that are not different from each other.
