# Supplementary material for: Persistent and sporadic Listeria monocytogenes strains do not differ when growing at 37 °C, in planktonic state, under different food associated stresses or energy sources
Source: BMC Microbiol. 2019 Nov 19;19:257. doi: 10.1186/s12866-019-1631-3 (PMC6862832; doi:10.1186/s12866-019-1631-3)
Supplement: Supplementary file 2 — Additional file 2: Figure S2. Box plots of average growth rate for L. monocytogenes isolates grown in chemically defined media (DM) at 25 mM concentrations of each energy source. Data includes averages of only replicates whose growth was observed (ΔOD600 ≥ 0.1), in log scale. Groups were created by use of Tukey’s HSD, where the same letters indicate means that are not different from each other. [file 12866_2019_1631_MOESM2_ESM.docx]

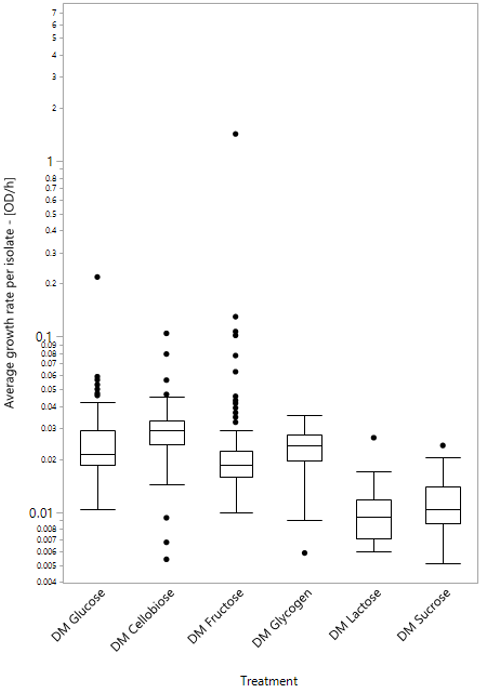


B

B

A

A

A

A

**Figure S2**: Box plots of average growth rate for *L. monocytogenes* isolates grown in chemically defined media (DM) at 25mM concentrations of each energy source. Data includes averages of only replicates whose growth was observed (ΔOD_600_ ≥0.1), in log scale. Groups were created by use of Tukey’s HSD, where the same letters indicate means that are not different from each other.
