# Supplementary material for: Persistent and sporadic Listeria monocytogenes strains do not differ when growing at 37 °C, in planktonic state, under different food associated stresses or energy sources
Source: BMC Microbiol. 2019 Nov 19;19:257. doi: 10.1186/s12866-019-1631-3 (PMC6862832; doi:10.1186/s12866-019-1631-3)
Supplement: Supplementary file 3 — Additional file 3: Table S1. Metadata of the 95 L. monocytogenes isolates analyzed. [file 12866_2019_1631_MOESM3_ESM.docx]

**Table S1**. Metadata of the 95 *L. monocytogenes* isolates analyzed

| Isolates | 96 Well Plate location | BioC Well | Persistence Factor | WGS-Corrected PFGE ^b^ | Store no.^a^ | SNP-Based Phylogenetic Clade |
| --- | --- | --- | --- | --- | --- | --- |
| FSL R8-5081 | A1 | 101 | Persistent | CU-SNP1 | 2 | U |
| FSL R8-5088 | A2 | 102 | Persistent | CU-SNP1 | 2 | U |
| FSL R8-5095 | A3 | 103 | Persistent | CU-258-69 | 7 | F |
| FSL R8-5230 | A4 | 104 | Persistent | CU-SNP3 | 2 | K |
| FSL R8-5233 | A5 | 105 | Persistent | CU-SNP1 | 2 | U |
| FSL R8-5449 | A6 | 106 | Persistent | CU-SNP3 | 2 | K |
| FSL R8-5487 | A7 | 107 | Sporadic | CU-57-267 | 7 | B |
| FSL R8-5543 | A8 | 108 | Sporadic | CU-SNP1 | 3 | V |
| FSL R8-5584 | A9 | 109 | Persistent | CU-11-326 | 8 | N |
| FSL R8-5805 | A10 | 110 | Persistent | CU-SNP2 | 23 | D |
| FSL R8-5797 | A11 | 111 | Persistent | CU-SNP1 | 18 | V |
| FSL R8-5844 | A12 | 112 | Persistent | CU-SNP2 | 23 | D |
| FSL R8-6046 | B1 | 113 | Persistent | CU-294-321 | 10 | A |
| FSL R8-6160 | B2 | 114 | Persistent | CU-SNP2 | 23 | D |
| FSL R8-6176 | B3 | 115 | Persistent | CU-SNP3 | 28 | I |
| FSL R8-6271 | B4 | 116 | Persistent | CU-SNP1 | 29 | V |
| FSL R8-6317 | B5 | 117 | Persistent | CU-SNP1 | 2 | U |
| FSL R8-6321 | B6 | 118 | Persistent | CU-57-267 | 10 | B |
| FSL R8-6446 | B7 | 119 | Persistent | CU-SNP1 | 18 | V |
| FSL R8-6480 | B8 | 120 | Persistent | CU-11-282 | 13 | M |
| FSL R8-6649 | B9 | 121 | Persistent | CU-SNP1 | 2 | U |
| FSL R8-6717 | B10 | 122 | Persistent | CU-57-267 | 10 | B |
| FSL R8-6721 | B11 | 123 | Persistent | CU-SNP1 | 10 | T |
| FSL R8-6765 | B12 | 124 | Persistent | CU-SNP2 | 23 | D |
| FSL R8-6836 | C1 | 125 | Sporadic | CU-SNP3 | 24 | I |
| FSL R8-7020 | C2 | 126 | Sporadic | CU-SNP1 | 12 | Q |
| FSL R8-7043 | C3 | 127 | Persistent | CU-57-267 | 10 | B |
| FSL R8-7057 | C4 | 128 | Sporadic | CU-294-321 | 7 | A |
| FSL R8-7121 | C5 | 129 | Persistent | CU-SNP1 | 29 | P |
| FSL R8-7153 | C6 | 130 | Persistent | CU-11-282 | 13 | M |
| FSL R8-7474 | C7 | 131 | Sporadic | CU-SNP1 | 11 | V |
| FSL R8-7493 | C8 | 132 | Persistent | CU-SNP1 | 29 | V |
| FSL R8-7534 | C9 | 133 | Persistent | CU-SNP1 | 16 | R |
| FSL R8-7554 | C10 | 134 | Sporadic | CU-SNP1 | 16 | S |
| FSL R8-7559 | C11 | 135 | Persistent | CU-262-318 | 21 | G |
| FSL R8-7585 | C12 | 136 | Persistent | CU-SNP2 | 23 | D |
| FSL R8-7599 | D1 | 137 | Persistent | CU-SNP2 | 23 | D |
| FSL R8-7716 | D2 | 138 | Persistent | CU-SNP1 | 29 | V |
| FSL R8-7722 | D3 | 139 | Persistent | CU-SNP3 | 29 | H |
| FSL R8-7761 | D4 | 140 | Persistent | CU-294-321 | 16 | A |
| FSL R8-7842 | D5 | 141 | Persistent | CU-SNP2 | 23 | D |
| FSL R8-7926 | D6 | 142 | Persistent | CU-182-173 | 19 | Y |
| FSL R8-8106 | D7 | 143 | Persistent | CU-8-340 | 8 | O |
| FSL R8-8110 | D8 | 144 | Persistent | CU-8-340 | 8 | O |
| FSL R8-8439 | D9 | 145 | Persistent | CU-258-69 | 7 | F |
| FSL R8-8453 | D10 | 146 | Persistent | CU-SNP1 | 16 | R |
| FSL R8-8466 | D11 | 147 | Persistent | CU-SNP3 | 2 | K |
| FSL R8-8477 | D12 | 148 | Sporadic | CU-SNP3 | 21 | I |
| FSL R8-8481 | E1 | 149 | Sporadic | CU-SNP3 | 21 | I |
| FSL R8-8505 | E2 | 150 | Persistent | CU-SNP1 | 24 | S |
| FSL R8-8509 | E3 | 151 | Persistent | CU-SNP1 | 2 | U |
| FSL R8-8514 | E4 | 152 | Persistent | CU-SNP3 | 2 | K |
| FSL R8-8523 | E5 | 153 | Persistent | CU-258-69 | 7 | F |
| FSL R8-8533 | E6 | 154 | Persistent | CU-SNP1 | 21 | Q |
| FSL R8-8665 | E7 | 155 | Persistent | CU-8-340 | 8 | O |
| FSL R8-8728 | E8 | 156 | Persistent | CU-SNP3 | 2 | K |
| FSL R8-8743 | E9 | 157 | Sporadic | CU-SNP1 | 7 | R |
| FSL R8-8744 | E10 | 158 | Sporadic | CU-SNP1 | 7 | R |
| FSL R8-8757 | E11 | 159 | Persistent | CU-SNP1 | 10 | T |
| FSL R8-8765 | E12 | 160 | Persistent | CU-SNP1 | 10 | T |
| FSL R8-8800 | F1 | 161 | Sporadic | CU-258-69 | 28 | F |
| FSL R8-8850 | F2 | 162 | Persistent | CU-SNP1 | 24 | S |
| FSL R8-8855 | F3 | 163 | Persistent | CU-SNP3 | 28 | I |
| FSL R8-8867 | F4 | 164 | Persistent | CU-55-266 | 2 | C |
| FSL R8-8876 | F5 | 165 | Persistent | CU-SNP3 | 2 | K |
| FSL R8-8881 | F6 | 166 | Persistent | CU-55-266 | 2 | C |
| FSL R8-8984 | F7 | 167 | Sporadic | CU-SNP1 | 29 | P |
| FSL R8-9141 | F8 | 168 | Persistent | CU-8-340 | 8 | O |
| FSL R8-9147 | F9 | 169 | Sporadic | CU-SNP1 | 29 | P |
| FSL R8-9298 | F10 | 170 | Sporadic | CU-SNP1 | 7 | R |
| FSL R8-9301 | F11 | 171 | Persistent | CU-258-69 | 7 | F |
| FSL R8-9308 | F12 | 172 | Persistent | CU-SNP1 | 10 | T |
| FSL R8-9314 | G1 | 173 | Sporadic | CU-SNP1 | 16 | S |
| FSL R8-9353 | G2 | 174 | Persistent | CU-55-266 | 2 | C |
| FSL R8-9354 | G3 | 175 | Persistent | CU-SNP1 | 2 | U |
| FSL R8-9374 | G4 | 176 | Persistent | CU-SNP1 | 16 | R |
| FSL R8-9383 | G5 | 177 | Persistent | CU-SNP1 | 21 | Q |
| FSL R8-9386 | G6 | 178 | Persistent | CU-SNP2 | 23 | D |
| FSL R8-9404 | G7 | 179 | Persistent | CU-SNP1 | 16 | R |
| FSL R9-0162 | G8 | 180 | Sporadic | CU-SNP1 | 7 | R |
| FSL R8-5646 | G9 | 181 | Sporadic | CU-258-69 | 22 | F |
| FSL R8-5726 | G10 | 182 | Sporadic | CU-258-69 | 27 | F |
| FSL R8-6637 | G11 | 183 | Persistent | CU-55-266 | 2 | C |
| FSL R8-6641 | G12 | 184 | Sporadic | CU-SNP1 | 2 | V |
| FSL R8-7161 | H1 | 185 | Persistent | CU-SNP1 | 13 | P |
| FSL R8-7348 | H2 | 186 | Sporadic | CU-294-321 | 2 | A |
| FSL R8-7825 | H3 | 187 | Persistent | CU-SNP2 | 23 | D |
| FSL R8-7833 | H4 | 188 | Persistent | CU-SNP3 | 23 | I |
| FSL R8-8485 | H5 | 189 | Persistent | CU-SNP1 | 24 | S |
| FSL R8-8495 | H6 | 190 | Persistent | CU-SNP3 | 28 | I |
| FSL R8-8778 | H7 | 191 | Persistent | CU-SNP1 | 21 | Q |
| FSL R8-8789 | H8 | 192 | Persistent | CU-SNP2 | 23 | D |
| FSL R8-9272 | H9 | 193 | Persistent | CU-SNP1 | 10 | T |
| FSL R8-9285 | H10 | 194 | Persistent | CU-SNP1 | 2 | U |
| FSL R9-0165 | H11 | 195 | Persistent | CU-SNP1 | 24 | S |
| BLANK | H12 | 196 | -- | -- | -- | -- |
| BLANK | -- | 197 | -- | -- | -- | -- |
| BLANK | -- | 198 | -- | -- | -- | -- |
| BLANK | -- | 199 | -- | -- | -- | -- |
| EMPTY | -- | 200 | -- | -- | -- | -- |

^a^ – Location number where the isolates were found

^b^ – ‘WGS Corrected PFGE’ indicates that isolates with certain PFGE patterns were found, in the source paper, to represent a single SNP-based phylogenetic clade. The presence or absence of a prophage likely caused the difference in PFGE pattern. Therefore, these were groups into a single SNP group: CU-SNP1, CU-SNP3, or CU-SNP3.
